# IFN-α as an Adjuvant for Adenovirus-Vectored FMDV Subunit Vaccine through Improving the Generation of T Follicular Helper Cells

**DOI:** 10.1371/journal.pone.0066134

**Published:** 2013-06-18

**Authors:** Chunxia Su, Xiangguo Duan, Jie Zheng, Lijun Liang, Feng Wang, Lin Guo

**Affiliations:** 1 School of Basic Medical Sciences, Ningxia Medical University, Yinchuan, Ningxia, China; 2 Laboratory examination college, Ningxia Medical University, Yinchuan, Ningxia, China; 3 General Hospital of Ningxia Medical University, Yinchuan, Ningxia, China; Federal University of São Paulo, Brazil

## Abstract

IFN-α exhibits either direct antiviral effects or distinct immunomodulatory properties, which was identified as a ‘natural immune adjuvant’ for both the innate and the adaptive immune responses. Here we have investigated the effects of IFN-α as an adjuvant on the generation of T follicular helper (Tfh) cells and antigen-specific antibody responses. The data showed that adenoviral vectors co-expressing FMDV VP1 proteins and porcine IFN-α potently enhanced the generation of Tfh cells, the secretion of IL-21 protein and the expression of Bcl-6 mRNA, compared with adenoviral vectors sole expressing VP1 alone. Additionally, IFN-α substantial increased the number of germinal-center (GC) B cells and formation of GCs. Furthermore, IFN-α enhanced the antibody response, as shown by increased production of all IgG and subclasses of IgG1 and IgG2a. Thus, our results revealed the potent adjuvant activity of IFN-α which enhanced the generation of Tfh cells and regulated the humoral immunity by promoting germinal-center reactions and antibody responses.

## Introduction

Type I interferons (IFN-I) form a gene family of more than 15 members in most mammals. Immunologists are mainly concerned with IFN-α (more than 10 genes) and IFN-β (one or two genes, depending on species). Originally identified as antiviral substances produced by infected cells, type I interferons (IFN-I) are now known to have a wide range of additional activities within both the innate and adaptive immune response [1]. Indeed, IFN-I was identified as ‘natural immune adjuvant’ for T and B cell-dependent acquired immunity [2]–[3]. The role of IFN-I as a natural immune adjuvant for commercial vaccines was established by showing that either mucosal or intramuscular administration of Inﬂuenza virus antigen-mixed IFN-I to mice [4]–[8].

In addition, some reports indicate that IFN-I induced immunomodulatory, as well as antiviral; activities may be involved in protection of swine against foot and mouse disease(FMD) [9]–[10].

Recent studies identified type I interferon as a natural adjuvant that selectively supports the generation of lymph node resident T follicular helper (Tfh) cells [11]–[12]. Tfh cells are a newly Th cell subset recently found to be present in germinal centers (GCs) and are characterized by their expression of chemokine (C-X-C motif) receptor 5 (CXCR5). These cells are thought to regulate humoral immunity, especially germinal-center reactions, helping antibody responses [13]–[17]. In addition to CXCR5, other markers have been also reported for Tfh cells, such as inducible costimulatory receptor (ICOS), programmed death-1 (PD-1) protein[18]–[19], IL-21 cytokine [20]–[22], and The B cell lymphoma 6 (Bcl6) transcription factor [23]. More recent studies have revealed that elevated expression of IL-21 and its receptor (IL-21R) distinguish Tfh cells from other Th subsets [24].

The presence of Tfh cells has been shown to be associated with increases in antibody production. Thus, understanding the antigen-specific mechanisms that control the differentiation of effector Tfh cells is critical for the design of future vaccines [25]–[26]. Furthermore, vaccine adjuvants that promoted responders of higher affinity also induced the largest number of antigen-specific Tfh cells in vivo [27]. Some studies shown that a malaria antigen with nanoparticle vaccines enhanced humoral responses correlated with enhanced GC formation and induction of antigen-specific Tfh cells [28].This study demonstrated that IFN-α as an adjuvant for adenovirus-vectored FMDV subunit vaccine through enhancement of humoral immunity by activating Tfh cells.

## Materials and Methods

### 2.1 Reagents and Animals

Female BALB/c mice were obtained from the Department of Experimental Animal, Ningxia Medical University, Yinchuan, Ningxia, China. This study was approved by the ethics committee of the Ningxia Medical University (Permit Date 2010/11). Mice were used at 4–6 weeks of age. RPMI medium 1640 was purchased from Gibco, fetal bovine serum (FBS) was purchased from Hyclone. AxyPrep Total RNA Miniprep Kit was purchased from AxyGEN. TransScript One-Step gDNA Removal and cDNA Synthesis SuperMix and TransStart Green qPCR SupermMix UDG were purchased from TransGen. rAd5VP1 is recombinant adenovirus(an E1/E3-deleted human adenovirus serotype 5) expressing foot and mouse disease virus (FMDV) VP1 protein [29], rAd5VP1-2A-PoIFN-α is recombinant adenovirus co-expressing FMDV VP1-2A genes and porcine IFN-α [30]. Mouse IL-21 ELISA Kit was purchased from Ray Biotech.

### 2.2 Antibodies

Alexa fluor@488 Rat Anti-Mouse CD4, APC Rat Anti-Mouse CXCR5, PE hamster Anti-Mouse PD-1, Alexa fluor@488 Rat Anti-Mouse B220, PE Rat Anti-Mouse GL-7 and Purified Rat Anti-Mouse CD16/CD32 (BD Pharmingen). Peanut Agglutinin-FITC (United States Biological). horseradish peroxidase-conjugated goat anti-mouse IgG, IgG1 and IgG2a (Sigma).

### 2.3 Immunization

4–6 week old female BALB/c mice were divided into 8 mice per group, each group was immunized with either 10^8^ TCID_50_ rAd5VP1 or 10^8^ TCID_50_ rAd5VP1-2A-PoIFN-α, and PBS was negative control. After seven days, mice were sacrificed for in vivo studies.

### 2.4 Flow Cytometric Analysis

Spleens from BALB/c mice were harvested on day 7 after rAd5VP1 or rAd5VP1-2A-PoIFN-α immunization, and splenocytes were analyzed by ﬂow cytometry to determine the frequency of CXCR5+PD-1+CD4+ Tfh cells, present within the total CD4+ population [18]–[19], and the frequency of GL-7+B220+ GC B cells, present within the total B220+ population [31]. Cells were washed and resuspended in PBS containing 1% FCS and 0.01% NaN3 (staining buffer). Cell surface staining was performed on ice with appropriate antibody dilutions and ﬂuorochrome combinations in staining buffer, after initial blocking of Fc-receptors with anti-CD16/CD32 (BD Pharmingen). mAbs included Alexa fluor@488 rat Anti-mouse CD4, APC rat Anti-mouse CXCR5, PE hamster Anti-mouse PD-1, Alexa fluor@488 rat Anti-mouse B220, PE rat Anti-mouse GL-7.Following a washing step in staining buffer. Data were collected on a FACS Aria? FCM B.D USA and analyzed using flowjo7.6.5 to assess which cell surface proteins were differentially expressed of different groups.

### 2.5 Immunofluorescence Microscopy

The spleens from BALB/c mice on day 7 after rAd5VP1 or rAd5VP1-2A-PoIFN-α immunization were fixed in 4% formalin and embedded in paraffin. Sections were cut to 4 µm thickness on a cryostat and stained for immunofluorescence using PNA-FITC for GCs according to the manufacturer’s instructions (United States Biological). All images were acquired on a BX51 OLYMPUS.

### 2.6 ELISA

Mice serum was harvested on day 7 after immunization.IL-21 was assayed by mouse IL-21 ELISA Kit according to the manufacturer’s instructions. The absorbance was measured at 450 nm using a spectrometer enzyme-labeled instrument (Bio-rad). We also assessed VP1-specific IgG, IgG1 and IgG2a antibodies in sera with ELISA and details protocol is described before [32]. In brief, mice serum was collected at 4 weeks after immunization. Recombinant VP1 protein (10 µg/ml in carbonate buffer pH 9.6) was coated overnight at 4°C. The plates were blocked with PBST containing 1% BSA for 2 hr at 37°C and then washed 3 times in PBS-Tween (0.05%).40-fold serial dilutions of sera in PBS-1% BSA were added to the wells for 2 hr at RT. After three washes, and antigen-specific antibodies of IgG, IgG1 and IgG2a were detected, and the OD490 was read by plate reader (Bio-Rad).

### 2.7 Quantitative Real-Time PCR

Splenocytes from BALB/c mice were harvested on day 7 after rAd5VP1 or rAd5VP1-2A-PoIFN-α immunization, and total cellular RNA was prepared with a AxyPrep Total RNA Miniprep Kit (AxyGEN), and then reverse transcribed using TransScript One-Step gDNA Removal and cDNA Synthesis SuperMix(TransGen), respectively, according to the manufacturer’s instructions. Bcl6 mRNA expression was assessed by real-time reverse transcription polymerase chain reaction (RT-PCR) analysis, which was performed using 20 ng cDNA template, TransStart Green qPCR SupermMix UDG (TransGen), and 0.5 mM of forward and reverse primers in a final volume of 25 µl. Reactions were run and recorded on an iCycler machine (Bio-Rad). Target genes were measured and normalized simultaneously with mouse β-actin endogenous controls. The following primer pairs for Bcl-6 and β-actin were used, Bcl-6 forward: 5′-CCC TGT GAA ATC TGT GGC ACT C-3′, reverse: 5′-ACA CGC GGT ATT GCA CCT TG-3′; β-actin: forward: 5′-GAT CTG GCA CCA CAC CTT CT-3′, reverse: 5′-ACC AGA GGC ATA CAG GGA CA-3′.

### 2.8. Statistics

All statistical tests were performed using Prism 5.The results were expressed as means±SD of the indicated number of experiments. And p values were calculated using t tests or 2 ways ANOVA, with a 95% confidence interval. *P<0.05; **P<0.01; ***P<0.001.

## Results

### 3.1 Adenoviral Vectors Co-expressing FMDV VP1 Proteins and IFN-α Enhances the Generation of Follicular Helper T Cells

**Figure 1:**
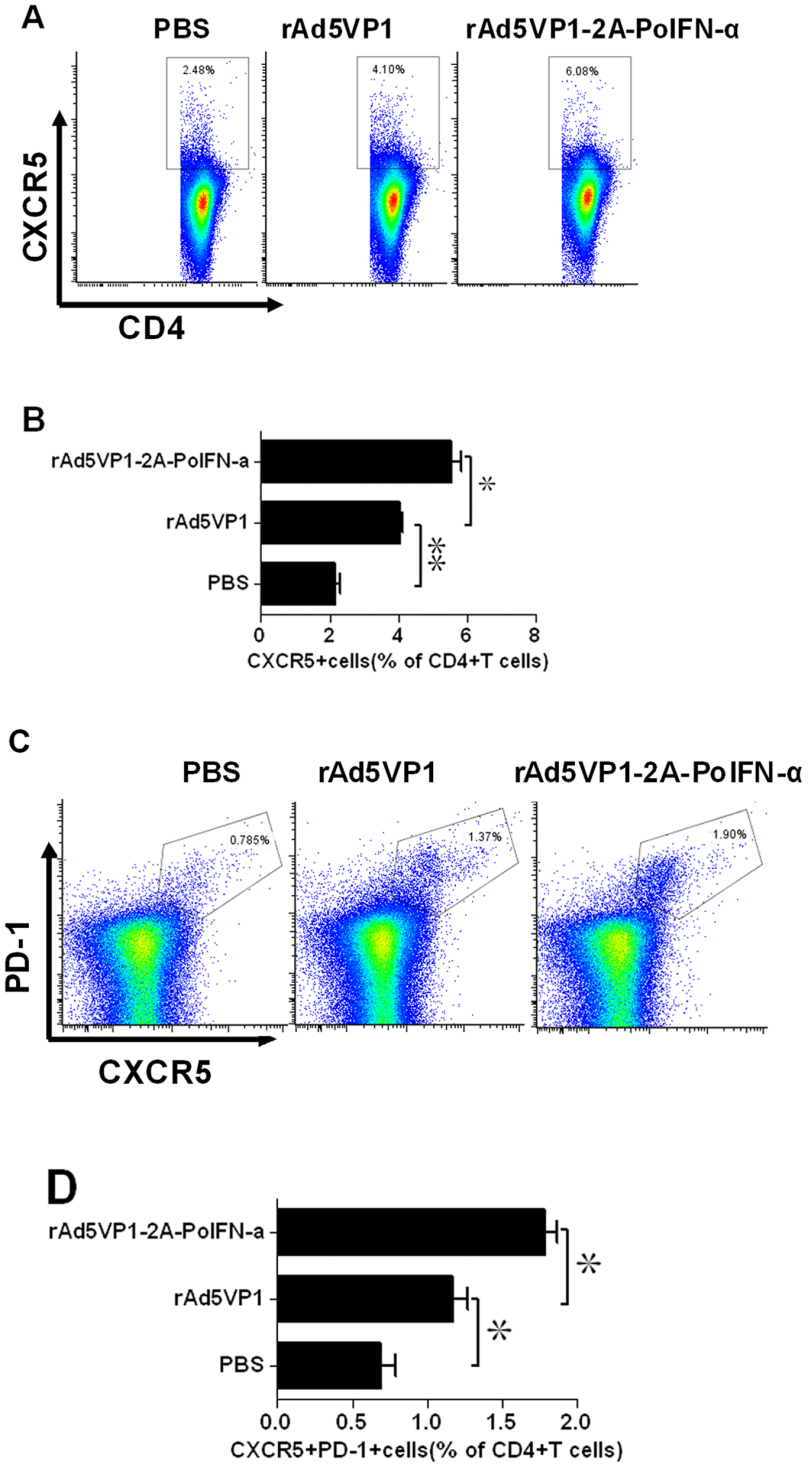
IFN-α enhanced the generation of Tfh cells. (**A–D**) Seven days after the immunization, Tfh cells were determined by staining with CD4, CXCR5,and PD-1 antibodies. (**A and C**) Representative dot plots from ﬂow cytometric analysis of Tfh cells. (**B and D**) Numbers in dot-plot quadrants represent the percentages in gated CD4+T.The graph shows means±SD.P values were calculated using a t test, *P<0.05, **P<0.01.

Tfh cells are characterized by increased expression of numerous molecules including the surface markers CXCR5 and PD-1. To investigate whether IFN-α up-regulates the expression of such molecules on Tfh cells, BALB/c mice were immunized with either adenoviral vectors expressing FMDV VP1 alone or co-expressing VP1 and IFN-α. The splenocytes were harvested on day 7 after immunization, and the development of CXCR5+PD-1+CD4+Tfh cells was determined by multiple-color ﬂow cytometry. Representative dot plot from ﬂow cytometric analysis of CD4+CXCR5+Tfh cells, as shown in Fig. 1A We examined substantial percentages of CD4+CXCR5+Tfh cells within total CD4+T cells and found a obvious increase in the frequency of CXCR5+CD4+T cells in mice immunized with adenoviral vectors co-expressing FMDV VP1 proteins and IFN-α(5.55±0.49%) compared with adenoviral vectors expressing VP1 alone(4.05±0.09%), as shown in Fig. 1B.

We next assessed whether increased expression of PD-1 is induced by co-expressing IFN-α. Representative dot plot from ﬂow cytometric analysis of PD1+CXCR5+Tfh cells, as shown in Fig. 1C. Mice immunized with vaccines developed substantial percentages of PD-1+CXCR5+Tfh cells within total CD4+T cells, rAd5VP1 (1.17±0.17%) and rAd5VP1-2A-PoIFN-α (1.78±0.13%). as shown in Fig. 1D. In addition to enhancing CXCR5 expression, we found that the expression of PD-1 was also greatly increased in mice immunized with adenoviral vectors co-expressing FMDV VP1 proteins and IFN-α.

**Figure 2:**
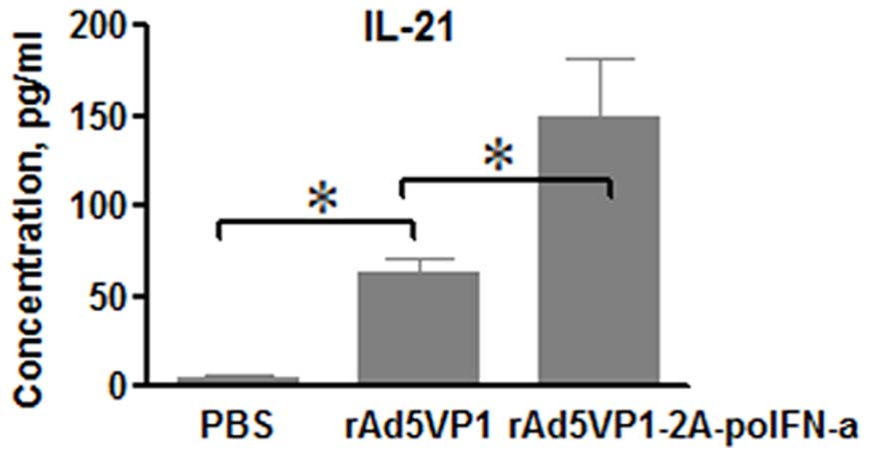
The production of IL-21 can be increased by IFN-α. IL-21 was measured by ELISA after 7 days of immunization. The graph shows means±SD.P values were calculated using a t test, *P<0.05; **P<0.01.

IL-21 is known to play an important role in Tfh differentiation and the development of B cell immunity in vivo [33]. To substantiate the above results, we also measured cytokine IL-21 secretion in mice serum after immunization by sandwich ELISA. Adenoviral vectors co-expressing FMDV VP1 proteins and IFN-α significant increased production of IL-21 protein, compared with adenoviral vectors expressing FMDV VP1 proteins alone (Fig. 2).

**Figure 3:**
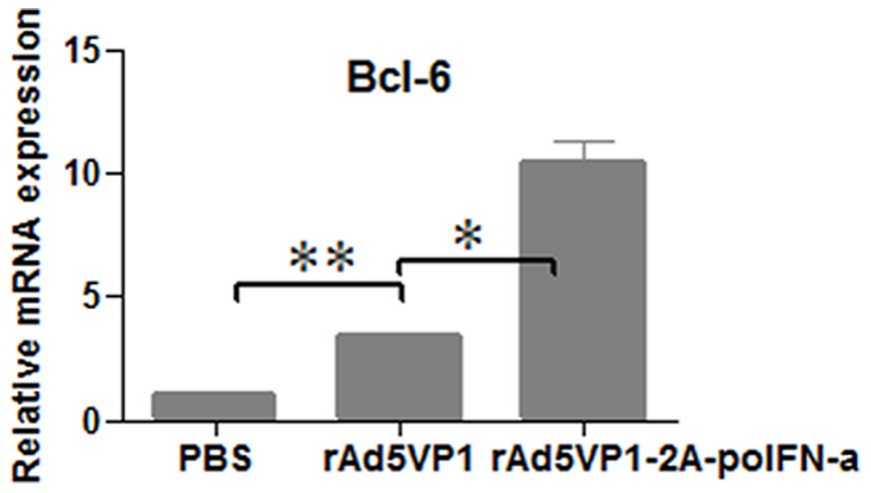
Bcl6 mRNA expression was analyzed by real-time RT-PCR. Seven days after the immunization, Bcl6 mRNA was measured and normalized simultaneously with mouse β-actin endogenous controls. The graph shows means±SD. P values were calculated using a t test, *P<0.05; **P<0.01.

The transcription factor Bcl6 is essential for the development of GC B cells and follicular helper T (Tfh) cells[34]–[35].We then assessed whether IFN-α increased Bcl6 expression by real-time RT-PCR. Consistent with above results, adenoviral vectors co-expressing FMDV VP1 proteins and IFN-α significant up-regulated Bcl6 expression, compared with adenoviral vectors expressing FMDV VP1 proteins alone (Fig. 3).

Overall, these data indicate that IFN-α greatly increased Tfh cell differentiation after immunized with adenovirus-vectored FMDV subunit vaccine.

### 3.2 Co-expression of FMDV VP1 Proteins and IFN-α Improved the Generation of GCs in Vaccinated Mice

**Figure 4:**
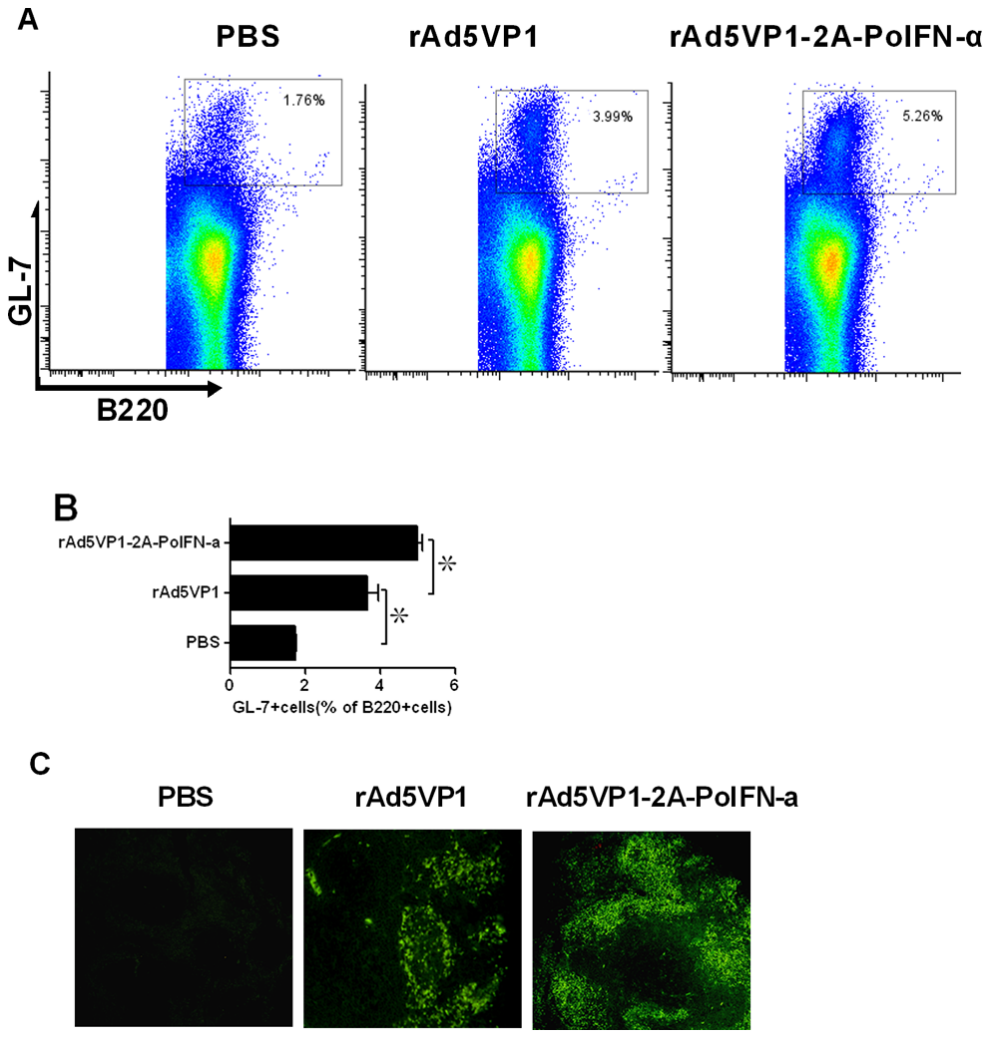
IFN-α enhanced the generation of germinal center B cells and the formation of GCs. Seven days after the immunization, germinal center B cells were determined by staining with GL-7 and B220 antibodies. (**A**) Representative dot plots from ﬂow cytometric analysis of GC B cells. (**B**) Numbers in dot-plot quadrants represent the percentages in gated B220+B cells. (**C**) GC in the spleens of vaccine -immunized mice were identified by PNA staining (green), The scale bar represents 80 µM.The graph shows means ±SD.P values were calculated using a t test, *P<0.05; **P<0.01.

Tfh cells are regarded as regulators of the germinal center reaction because they help to activated B cells. To investigate whether GC B cells were formation after vaccines immunization, Spleens were harvested on day 7, and splenocytes were analyzed by ﬂow cytometry to determine the frequency of B220+GL-7+ B cells, which have been defined as GC B cells [33]. Representative dot plot from ﬂow cytometric analysis of B220+GL-7+ B cells as shown in Fig. 4A. Consistently, we also observed the fraction of GL-7+B220+B cells was significantly increased in mice immunized with adenoviral vector co-expressing FMDV VP1 proteins and IFN-α(5.00±0.22%) compared with mice immunized with rAd5VP1 alone (3.66±0.53%), as shown in Fig. 4B. To confirm the above results, GCs were detected by histological stains of lymphoid tissue using the lectin PNA, which is a lectin that binds with nonsialylated core 1 O-glycans. Moreover, the generation of GCs is greatly improved in mice immunized with co-expression of FMDV VP1 proteins and IFN-α compared with mice immunized with rAd5VP1 alone, which was consistent with our ﬂow cytometric analysis (Fig. 4C).

### 3.3 Co-expression of FMDV VP1 Proteins and IFN-α Enhanced Antibody Titers

**Figure 5:**
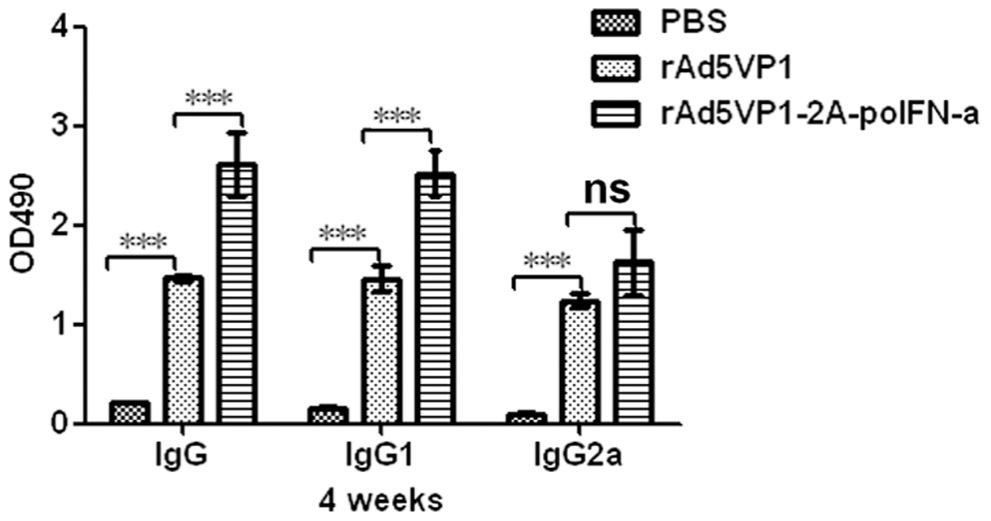
IFN-α enhanced the production of antigen-specific total IgG and IgG1 and IgG2a. 4 weeks after the immunization,FMDV VP1–specific total IgG and IgG1 and IgG2a were detected by ELISA, The graph shows means ±SD.P values were calculated using 2 way ANOVA, *P<0.05; **P<0.01; ***P<0.001.

We also demonstrated IFN-α as an adjuvant that enhances antibody responses for the specific FMDV VP1, 4 weeks after the immunization, the sera were assayed by ELISA for the presence of FMDV VP1-specific IgG, IgG1 and IgG2a antibodies. IFN-α stimulated a marked increase in the FMDV VP1-specific antibody titers, especially in IgG antibodies and IgG1 subclass (Fig. 5).

## Discussion

Type I IFN possesses strong adjuvant activity, markedly enhancing the antibody response against a poorly immunogenic soluble protein. Some studies showed type I IFN clearly enhances T cell-dependent Ab responses by direct signaling in T and B cells[36]-[37]. Amongst Th cells, T follicular helper (Tfh) cells are specialized in promoting protective, long-lived antibody responses that arise from germinal centers. Our findings support a correlation vaccines enhanced humoral responses and enhanced GC formation and induction of antigen-specific Tfh cells.

In this study, we used adenoviral vector co-expressing FMDV VP1 and IFN-α and adenoviral vector expressing FMDV VP1 alone as vaccines to immunize mice, we observed generation of CXCR5+CD4+Tfh cells after rAd5Vp1 and rAd5VP1-2A-PoIFN-α immunization, and IFN-α greatly enhanced the differentiation of CXCR5+CD4+Tfh cells. Some study demonstrated that IFN-α directly promotes PD-1 transcription [38]; consistently, we also observed increased percentages of CXCR5+PD-1+cells after rAd5VP1-2A-PoIFN-α immunization, compared with rAd5Vp1 alone.

To determine whether rAd5VP1-2A-PoIFN-α had an effect on the production of IL-21, which plays an important role in Tfh differentiation and the development of B cell immunity in vivo [33]. Our ELISA result showed that rAd5VP1-2A-PoIFN-α could enhance IL-21 production, compared with rAd5Vp1 alone.

Bcl6 is selectively expressed by Tfh cells [15]. A current study indicated that Bcl6 promotes the expression of Tfh-related genes but inhibits the differentiation of Th1, Th2, and Th17 cells and supported Bcl6-dependent Tfh cell generation as a pathway that is independent of other Th cell lineages [24], [35]. In this study Bcl6 mRNA expression was assessed by Real-Time PCR analysis, consistently, The Bcl6 mRNA expression was increased by rAd5VP1-2A-PoIFN-α.

Some studies reveal that the failure of IFN-αR1−/− mice to efficiently support Tfh cell differentiation and accumulation within B cell follicles is linked with an impaired Ab affinity maturation, which showing that IFN-α promotes GC formation and is critical for production of neutralizing Abs following adenoviral infection [12], [37]. Thus, we examined GCs formation and GC B cells differentiation following rAd5VP1 and rAd5VP1-2A-PoIFN-α immunizations. The formation of GCs and the percentage of GL-7+B220+GC B cells were increased by rAd5VP1-2A-PoIFN-α.

Type I IFN augmented the production of all subclasses of IgG during the primary antibody response and induced both long-term antibody production and immunological memory after a single injection of soluble protein [8], consistently, the production of total IgG, IgG1 and IgG2a were increased by rAd5VP1-2A-PoIFN-α. Thus, IFN-α exhibited a significant adjuvant effect in mice inducing generation of Tfh cells.

In summary, IFN-α enhanced humoral response correlated with enhanced GC formation and induction of antigen-specific Tfh cells, suggesting it utility in recombinant vaccination as an adjuvant. Thus, IFN-α adjuvant may be a promising strategy to enhance the durability, breadth, and potency of humoral immunity by enhancing key elements of Tfh cells and the B-cell response.
